# Incidence and cost of hospitalizations associated with *Staphylococcus aureus* skin and soft tissue infections in the United States from 2001 through 2009

**DOI:** 10.1186/1471-2334-14-296

**Published:** 2014-06-02

**Authors:** Jose A Suaya, Robertino M Mera, Adrian Cassidy, Patrick O’Hara, Heather Amrine-Madsen, Stuart Burstin, Loren G Miller

**Affiliations:** 1 Health Outcomes, North America Vaccine Development, GlaxoSmithKline, Philadelphia, PA, USA; 2 Gastroenterology Department, Vanderbilt University, Nashville, TN, USA; 3 GSK Vaccines, Wavre, Belgium; 4 Research and Development, GlaxoSmithKline, Philadelphia, PA, USA; 5 Research and Development, GlaxoSmithKline, RTP, Durham, NC, USA; 6 North America Vaccine Development, GlaxoSmithKline Vaccines, King of Prussia, PA, USA; 7 Los Angeles BioMedical Research Center at Harbor-UCLA Medical Center; Division of Infectious Diseases, David Geffen School of Medicine at UCLA, Los Angeles, CA, USA

**Keywords:** *Staphylococcus aureus*, Skin and soft tissue infections (SSTIs), Hospitalizations, Incidence, Cost

## Abstract

**Background:**

The emergence of community-associated methicillin-resistant *Staphylococcus aureus (SA)* and its role in skin and soft tissue infections (SSTIs) accentuated the role of *SA*-SSTIs in hospitalizations.

**Methods:**

We used the Nationwide Inpatient Sample and Census Bureau data to quantify population-based incidence and associated cost for *SA*-SSTI hospitalizations.

**Results:**

*SA*-SSTI associated hospitalizations increased 123% from 160,811 to 358,212 between 2001 and 2009, and they represented an increasing share of *SA-* hospitalizations (39% to 51%). *SA*-SSTI incidence (per 100,000 people) doubled from 57 in 2001 to 117 in 2009 (p < 0.01). A significant increase was observed in all age groups. Adults aged 75+ years and children 0–17 years experienced the lowest (27%) and highest (305%) incidence increase, respectively. However, the oldest age group still had the highest *SA*-SSTI hospitalization incidence across all study years. Total annual cost of *SA*-SSTI hospitalizations also increased and peaked in 2008 at $4.84 billion, a 44% increase from 2001. In 2009, the average associated cost of a *SA*-SSTI hospitalization was $11,622 (SE = $200).

**Conclusion:**

There has been an increase in the incidence and associated cost of *SA*-SSTI hospitalizations in U.S.A. between 2001 and 2009, with the highest incidence increase seen in children 0–17 years. However, the greatest burden was still seen in the population over 75 years. By 2009, SSTI diagnoses were present in about half of all *SA-*hospitalizations.

## Background

Skin and soft tissue infections (SSTIs) are common reasons for seeking medical care, and can have significant morbidity including hospitalization [1,2]. In patients hospitalized due to SSTI, *Staphylococcus aureus* (*S. aureus*) is the most common bacterial pathogen identified [3-5].

In the past, methicillin-resistant *S. aureus* (MRSA) infections were mostly confined to patients with risk factors such as hospitalization or recent healthcare contact [6]. More recently however, there has been a dramatic increase in community-associated MRSA (CA-MRSA) infections which often present as SSTIs, affecting individuals without prior risk factors^3;6^. The emergence of MRSA in SSTIs has challenged treatment, as this bacterium is resistant to beta-lactam antibiotics that were previously the standard of care for *S. aureus*-SSTI treatment [5].

In the U.S.A. between 1997 and 2005, the overall annual rate of visits for SSTIs to physician offices, hospital outpatient departments, and emergency departments increased from 32.1 to 48.1 visits per 1,000 people, which at the U.S. population level, equated to 14.2 million total SSTI visits in 2005 [7]. *S. aureus* hospitalizations also increased in parallel with the increase in SSTIs. Between 1999 and 2005, the estimated number of *S. aureus*–related hospitalizations across infection types increased by 62%, from 294,570 to 477,927 and the estimated number of MRSA-related hospitalizations more than doubled, from 127,036 in 1999 to 278,203 in 2005 [8]. The increase in *S. aureus* hospitalizations has occurred coincidentally with the emergence of the USA300 clone as a primary cause of CA-MRSA infections, which mostly manifest as SSTIs [9].

In terms of management of *S. aureus*-SSTIs, treatment costs can be substantial. Cost estimates vary by populations studied, cost perspective, and antibiotic therapy chosen, among other factors [10,11]. In a U.S.A. study of 1997 inpatients and outpatients, Marton, et al., reported that mean overall SSTI episode costs among adults were $8,865 (with 12.6% of the episodes resulting in hospitalization) [10]. Menzin, et al., studied over 13,000 *S. aureus*-SSTI inpatient episodes from 2005 to 2006 and found that the mean associated hospitalization costs were $6,800 per patient [12]. Itani, et al., studied 5,156 patients hospitalized with SSTIs and found that the median charge among the 3,079 with gram positive-associated SSTIs (which includes *S. aureus*) was $19,894 with a mean of $40,046 [2].

The above investigations were limited by relatively small sample size and/or relatively short study time frames. To address these data limitations, we performed a retrospective observational study to evaluate the US national trend of the incidence and cost of hospitalizations associated to *S. aureus*-SSTIs from 2001 through 2009.

## Methods

### Data sources

The main data source was the Healthcare Cost and Utilization Project (HCUP) Nationwide Inpatient Sample (NIS) of the Agency for Healthcare Research and Quality, which is the largest U.S. database of inpatient stays representing approximately 20% of all-payer discharges occurring in U.S.A. community hospitals annually. Data on individual hospitalizations used in this survey include patient demographics, clinical and procedure diagnoses, length of stay, total charges, and cost-to-charges ratios [13]. The study period was from 2001 through 2009. To obtain national representative estimates, all analyses used sample weights and variance calculations as provided by HCUP. These weights were used to account for the complex sample design of the NIS data such as unequal probabilities of selection, oversampling, and non-responses [13].

### Ethics statement

This study was conducted in strict adherence to the HCUP Data Use Agreement (DUA) with the Healthcare Cost and Utilization Project Agency for Healthcare Research and Quality to protect the privacy rights of the individuals and institutions within the HCUP data sets. No attempts were made to identify patients, physicians, and other health care providers. The study design was reviewed by the Compliance Office of the Los Angeles Biomedical Research Institute at Harbor-UCLA Medical Center and determined to be exempt from regulatory requirement of human subject’s research.

### Annual incidence of hospitalizations associated with *S. aureus*-SSTIs

We used the International Classification of Diseases, 9th Revision, Clinical Modification (ICD-9-CM) in any of up to the first seven hospital discharge diagnoses to identify hospital discharges of interest. To estimate unadjusted, overall and age-specific cumulative incidence rates we followed three steps. First, we identified all hospitalizations with the diagnoses of *S. aureus* infections and among them, those hospitalizations with concomitant diagnosis of SSTI. Specifically, we used the codes for *S. aureus* septicaemia (0.38.11 and 038.12), *S. aureus* infection (041.11 and 041.12), and *S. aureus* pneumonia (482.41 and 482.42) to identify any *S. aureus* infection. It is noteworthy to report that some ICD-9 codes for *S. aureus* infections were not available during the entire study period but were added as new codes in October 2008 (i.e., 038.12, 041.12, and 482.42) [14]. Based on previously published investigations, we used the following codes for the identification of SSTI-related hospitalizations: Carbuncle (680.xx); Cellulitis and abscesses (681.xx and 682.xx); Erysipelas (035.xx); Impetigo (684.xx); Mastitis (611.0.x and 771.5×); Other (7048.x), Pyoderma Dermatitis (686.xx); Pressure ulcer (707.xx); Infection due to internal prosthetic device, implant, & graft (996.6×); Postoperative infection (998.5×); Other infection, as complication of medical care (9993.x), or Non-healing surgical wound (998.83) [8-14]. For descriptive purposes, hospitalizations were stratified by patient age group (0–17, 18–44, 45–64, 65–74 and 75 years and older).

We then used annual overall and age-group population estimates for 2001 through 2009 from the U.S.A. Census Bureau [15]. We divided the annual number of hospitalizations of interest by the annual population estimates and reported annual cumulative incidences as hospitalizations per 100,000 people. We then calculated the proportion of all *S. aureus* hospitalizations with concomitant diagnosis of SSTI. Finally, we graphically assessed incidence trends over time for the point estimates and their 95% confidence intervals.

### Average length of stay and average cost associated with *S. aureus*-SSTIs hospitalizations

For each hospitalization of interest, we calculated the length of stay and the hospital cost of medical care. As hospital charges are usually not reimbursed in full, we converted charges into cost (which are lower than charges), by using HCUP cost-to-charge ratios [16]. All costs were then converted into 2010 US dollars by using the Consumer Price Index for Medical Care, Hospital, and Related Services [17].

### Annual national cost associated with *S. aureus*-SSTIs hospitalizations

For each study year we calculated the total national cost associated with *S. aureus* and *S. aureus*-SSTI hospitalizations by multiplying the number of hospitalizations by their average cost. Cost estimates were also calculated as a proportion of all *S. aureus* hospitalizations (i.e., non-SSTI infections such as bloodstream infections and pneumonia). We graphically assessed annual cost trends over time for the point estimates and their 95% confidence intervals.

### Statistical Methods

We quantified relative changes in incidence, length of stay, average cost per stay and national cost of *S. aureus*-SSTI hospitalizations between the first and last year of the study period (2001–2009). We assessed statistical significance at an *a priori* alpha level of 0.05. The use of HCUP’s sample weights and variance was described above. All analyses were performed in SAS software, version 9.1 (SAS Institute, Cary North Carolina).

## Results

**Figure 1:**
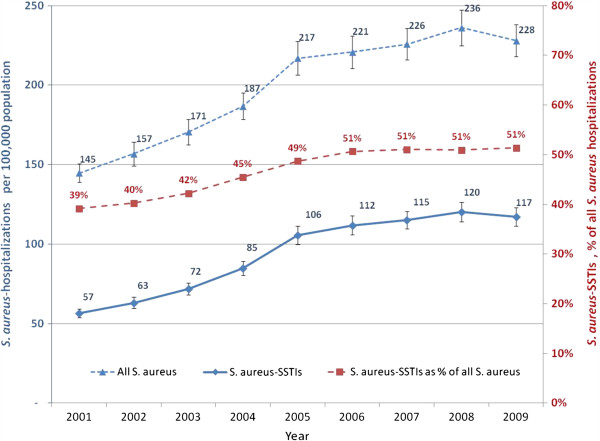
**Incidence of *S. aureus *** **hospitalizations overall and those associated with skin and soft tissue infection (SSTI) in U.S.A., 2001–2009.**

From 2001 through 2009, hospitalizations associated with any *S. aureus* infection increased from 410,768 to 697,248 hospitalizations, a 70% increase (p < 0.001). During our study period, the peak year for *S. aureus* and *S. aureus*-SSTI hospitalizations was 2008, with a subsequent non-significant decrease in 2009. During the same period, the total number of hospitalizations for any reason increased non-significantly by 6% (37.2 to 39.4 million). Among all *S. aureus* associated hospitalizations, SSTIs comprised an increasing proportion over time (39% in 2001 to 51% in 2009, p < 0.01) (Table 1 and Figure 1). Whilst the incidence of any *S. aureus* hospitalization increased by 57% between 2001 and 2009 (from 145 to 228 per 100,000, p < 0.01), the incidence of *S. aureus*-SSTIs increased by 105% in the same period (from 57 to 117 per 100,000, p < 0.01) (Figure 1).

**Table 1:** **Hospitalizations for *S. aureus * skin and soft tissue infections (SA-SSTI) in the U.S. by year and age group, 2001–2009**

**Year**																		
	**2001**		**2002**		**2003**		**2004**		**2005**		**2006**		**2007**		**2008**		**2009**	
**Description**	**Number**	**SE**	**Number**	**SE**	**Number**	**SE**	**Number**	**SE**	**Number**	**SE**	**Number**	**SE**	**Number**	**SE**	**Number**	**SE**	**Number**	**SE**
All SA hospitalizations	410,768	8,489	449,232	11,032	493,199	11,636	545,022	12,530	6389,856	15,794	656,181	15,532	677,580	15,104	715,596	17,397	697.248	15628
All SA-SSTIs hospitalizations	160,811	3,910	180,748	5,170	208,219	5,579	247,821	6,510	310,982	8,861	332,378	8,989	345,706	8,249	364,452	9,677	358,212	9,143
SA-SSTIs hospitalizations by age-group																		
0-17 years	8,191	908	11,759	2,397	14,806	1,570	20,865	2,167	36,771	4,614	33,373	2,714	35,622	2,969	38,289	3,581	34,076	2,697
18-44 years	33,480	1,115	40,838	1,623	51,773	1,864	68,493	2,318	89,811	3,459	99,431	3,500	98,636	2,975	99,368	3,263	93,018	2,799
45-64 years	48,857	1,334	57,117	1,683	66,325	,1825	79,827	2,287	96,897	3,020	107,279	3,123	113,515	3,128	120,949	3,444	124,924	3,501
65-74 years	26,737	830	27,662	875	29,014	893	31,535	946	34,956	1,149	37,784	1,099	40,219	1,058	43,720	1,193	44,585	1,283
75 plus years	43,535	1,355	43,368	1,429	46,275	1,483	46,993	1,407	52,470	1,748	54,449	1,730	57,666	1,638	61,999	1,865	61,322	1,847

Notes.

Data source: The Nationwide Inpatient Sample, Healthcare Cost and Utilization Project (HCUP), Agency for Healthcare Research and Quality.

SA-SSTIs hospitalizations were defined by any *S. aureus* infection codes concurrent with any skin and soft tissue infection codes. *S. aureus* hospitalizations were identified based on the following ICD-9-CM codes: *S. aureus* septicemia (0.38.11 and 038.12), *S. aureus* infection (041.11 and 041.12), and *S. aureus* pneumonia (482.41 and 482.42). Some ICD-9 codes for *S. aureus* infections were not available during the entire study period but added as new codes in October 2008 (i.e., 038.12, 041.12, and 482.42).

Skin and soft tissue infections were identified based on the following ICD-9-CM codes: carbuncle (680.xx), cellulitis and abscesses (681.xx and 682.xx), erysipelas (035.xx), impetigo (684.xx), mastitis (611.0.x and 771.5×), other (7048.x, pyoderma dermatitis (686.xx), pressure ulcer (707.xx), infection due to internal prosthetic device, implant, & graft (996.6×), Postoperative infection (998.5×), other infection; as complication of medical care (9993.x), or non-healing surgical wound (998.83).

SA-SSTIs hospitalizations were defined by any *S. aureus* infection codes concurrent with any skin and soft tissue infection codes.

**Figure 2:**
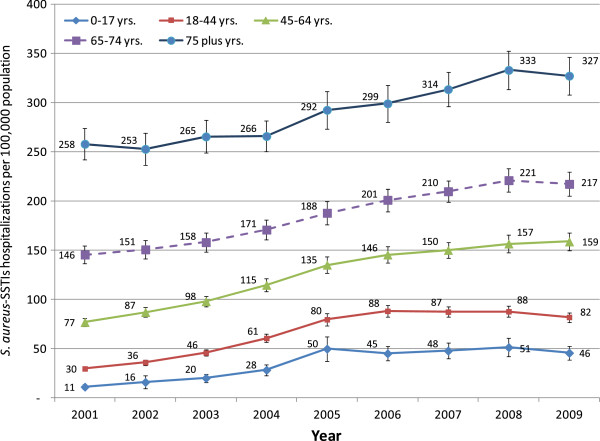
**Incidence of *S. aureus*-skin and soft tissue infection (SSTI) hospitalizations by age group in U.S.A., 2001–2009.**

The incidence of *S. aureus*-SSTIs varied by age-groups, with the highest incidence among those >75 years (327 per 100,000) and the lowest incidence among those 0–17 years of age (46 per 100,000) (Figure 2). All age groups had an increased incidence of hospitalizations, with many age groups displaying a peak incidence in 2008 (Figure 2). While older age groups had higher incidence in all the years than younger age groups, the greatest relative growth in incidence occurred in younger populations. Persons age 0–17 years had a 318% increase in *S. aureus*-SSTIs during the study period (p < 0.01) and adults aged 18–44 years had a 173% increase (p < 0.01). Despite these dramatic increases in the youngest age group, the incidence of *S. aureus*-SSTI hospitalizations in people age 75 years and above was still approximately seven times of that in children 0–17 years.

**Figure 3:**
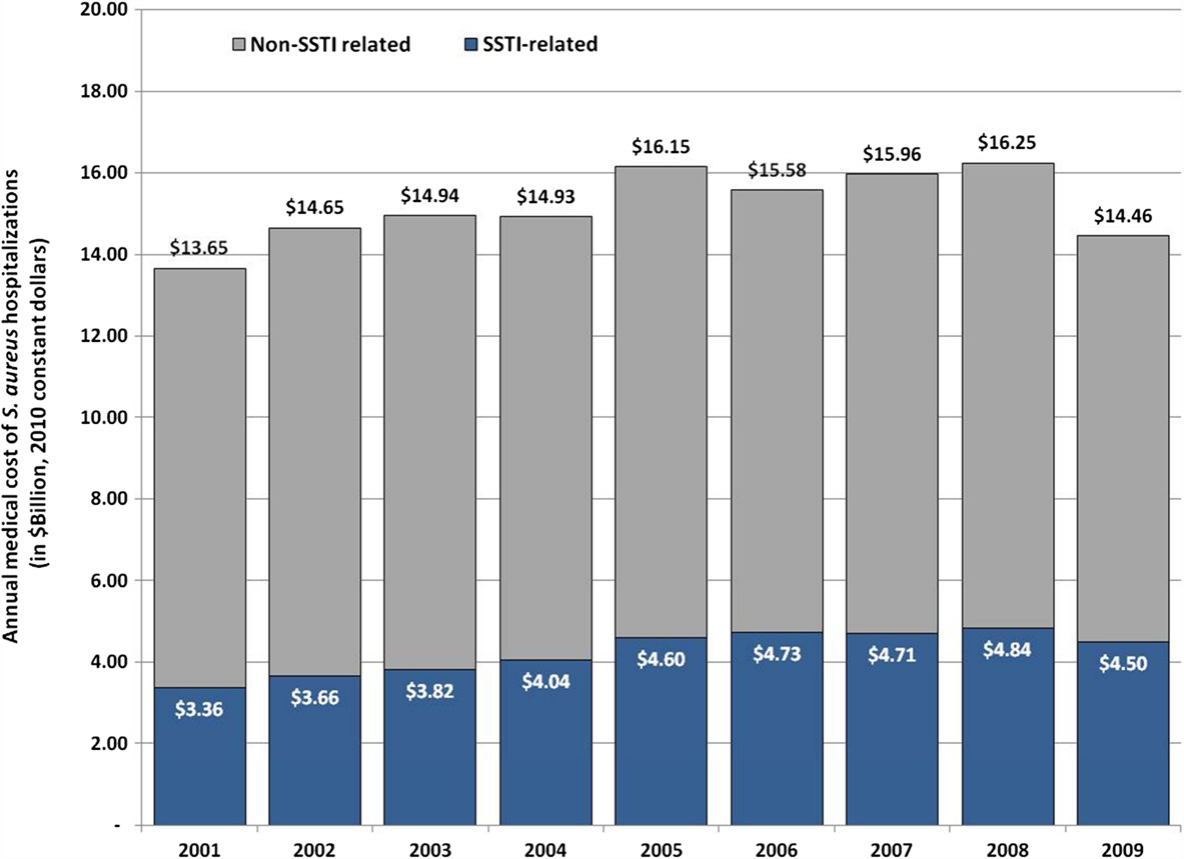
**Annual medical associated cost of *S. aureus *** **hospitalizations and role of skin and soft tissue infections (SSTIs) in U.S.A., 2001–2009.**

There was a moderate yet significant increase in the national annual cost of *S. aureus*-hospitalizations between 2001 and 2009 from $13.65 billion to $14.46 billion, a 6% increase (p < 0.01), with a peak of $16.25 billion in 2008 (Figure 3). However, during the same period, the national cost of *S. aureus*-SSTI hospitalizations experienced a much more pronounced increase (34% increase, p < 0.01), from $3.36 billion to $4.50 billion, peaking in 2008 at $4.84 billion (Figure 3). Thus during our study period *S. aureus*-SSTI hospitalizations accounted for an increasingly larger share of cost associated with *S. aureus* hospitalizations (from 25% to 31%, p <0.01).

The observed temporal variations in national costs were driven by a number of factors that acted in different directions. First, average length of stay and the average cost of hospitalization for *S. aureus* associated SSTIs decreased. This trend was counterbalanced by the higher incidence of *S. aureus*-SSTI hospitalizations. Table 2 describes these changes between 2001 and 2009 overall, and stratified by age groups. For example, between 2001 and 2009, in children 0–17 years of age, there was a reduction of 43%, in the average length of hospital stay (8.1 to 4.7 days, p < 0.05), and a reduction of 66% in the average cost of a hospitalization ($22,601 to $7,740, p < 0.05). However, these changes were counterbalanced by a 305% (11.29 to 45.74 per 100,000, p < 0.05) increase in hospitalization incidence. In combination, these forces resulted in a 61% increase ($160 to $260 million, p < 0.05) in the national cost of *S. aureus*-SSTI hospitalizations within this age group during the study period. In persons age ≥ 75 years, while there was a 17% (10.7 to 8.9 days, p < 0.05) decrease in the length of stay, a 35% decrease ($20,587 to $13,346, p < 0.05) in the average cost per hospitalization, and a 27% increase (258 to 327 per 100,000, p < 0.05) in the incidence of hospitalizations, the overall impact in the annual cost was a 10% decrease ($930 to $830 million, not significant).

**Table 2:** **Length of stay, average cost, incidence and cost changes of hospitalizations associated with *S. aureus *** **skin and soft tissue infections (SA-SSTI) by age group in the U.S**.

	**Year**				**Period change**
	**2001**		**2009**		**2001–2009**
**Item**	**Estimate**	**SE**	**Estimate**	**SE**	**% Change**
SA-SSTI length of stay of hospitalization					
All ages	9.91	0.16	7.26	0.11	-27%*
0–17 yrs.	8.14	0.67	4.65	0.23	-43%*
18–44 yrs	8.31	0.19	5.91	0.10	-29%*
45–64 yrs.	10.00	0.22	7.65	0.11	-23%*
65–74 yrs.	11.03	0.25	8.82	0.18	-20%*
75 plus yrs.	10.71	0.19	8.85	0.18	-17%*
SA-SSTI average cost of hospitalization					
All ages	21,287	641	11,622	200	-45%*
0–17 yrs.	22,601	2,881	7,740	887	-66%*
18–44 yrs.	18,675	738	9,309	214	-50%*
45–64 yrs.	22,379	784	12,539	213	-44%*
65–74 yrs.	23,206	795	14,456	278	-38%*
75 plus yrs.	20,587 670		13,346	231	-35%*
SA-SSTI hospitalizations per 100,000 population					
All ages	56.68	1.38	117	2.99	+107%*
0–17 yrs.	11.29	1.25	45.74	3.62	+305%*
18–44 yrs.	29.77	0.99	82.03	2.47	+176%*
45–64 yrs.	77.00	2.10	158.84	4.45	+106%*
65–74 yrs.	145.57	4.52	217.29	6.25	+49%*
75 plus yrs.	258.01	8.03	327.20	9.86	+27%*
National annual cost of SA-SSTI hospitalizations (in $ Billion)					
All ages	3.36	0.16	4.22	0.14	+26%*
0–17 yrs.	0.16	0.03	4.22	0.14	+61%*
18–44 yrs	0.58	0.04	0.88	0.03	+51%*
45–64 yrs.	1.07	0.06	1.59	0.06	+49%
65–74 yrs.	0.62	0.04	0.66	0.02	+5%
75 plus yrs.	0.93	0.05	0.83	0.03	-10%

Note: cost estimates are in constant 2010 US dollar. Asterisk (*) denotes statistical significant differences at alpha = 0.05.

## Discussion

Based on HCUP data, which is comprised of 20% of all US hospitalizations between 2001 and 2009, we found an increase in *S. aureus* associated hospitalizations consistent with prior reports [8,9,18]. Importantly, we found that among *S. aureus* infection the proportion of hospitalizations with a diagnosis code for SSTI also increased, indicating that the rise in *S. aureus* infections was at least in part driven by SSTIs. By 2009, half of all *S. aureus* -hospitalizations were associated with a SSTI code.

We also found that between 2001 and 2009 costs associated with *S. aureus*-SSTI hospitalizations increased 26%, which would imply an increase of national cost from $3.36 to $4.22 billion in constant dollars. Trends in *S. aureus*-SSTI hospitalization cost increases may be explained, at least in part, by changes in the age group demographics of hospitalization, hospital length of stay, and/or average hospitalization cost. For example, by 2009, patients aged 0–44 years accounted for a larger proportion of hospitalizations than in 2001 (36% vs. 26%, respectively). Furthermore, the average length of stay and cost of hospitalization dropped within these age groups more than in the older age strata (Table 2). Therefore, the fact that the national annual cost of *S. aureus*-SSTI hospitalizations did not increase even higher was because the higher *S. aureus*-SSTI hospitalization rates were attenuated by increasingly lower age of those hospitalized (who have less expensive hospitalizations) as well as shorter length of stay in all age groups over time. We found a mean cost of *S. aureus*-SSTI of $11,622 in 2009, which falls into the cost/charge range found in the literature from $6,800 to $40,046 [12].

The increase in SA-SSTIs seen disproportionately in the younger age groups has occurred at a time of an increased incidence of infections due to CA-MRSA caused by the USA 300 strain, which has also been noted in the younger age groups [18,19].

Our findings on the increased incidence of *S. aureus-*SSTI hospitalizations are generally in agreement with prior studies. Klein, et al., showed a 62% increase in the incidence of *S. aureus* hospitalization between 1999 and 2005 [8], which is similar to the 55% increase between 2001 and 2005 we have found. Interestingly, there are recent data demonstrating decreases in invasive MRSA infections between 2005 and 2011 [20]. However, we did not see decreases, most likely because the decreases noted by Dantes, et al., appear to be driven largely by decreases in healthcare-associated MRSA infections, likely due to hospital-based infection prevention efforts [20], whereas our data focused on SSTIs, which are typically community associated and thus less likely to be affected by hospital-based prevention efforts.

Our study has several limitations. First, we calculated the estimated costs of *S. aureus*-SSTIs using associated cost estimates, rather than excess cost estimates. Thus, we have likely overestimated the cost of *S. aureus* associated SSTIs because our analysis includes the costs of concomitant treatment of comorbidities. On the other hand, we may have underestimated the *S. aureus*-SSTI associated hospitalization cost because HCUP provides information on direct hospital costs only, and not costs associated with physicians and other medical professionals that provided care but which are billed separately. Second, diagnoses of *S. aureus*-SSTI were based solely on ICD-9 codes and were not validated with chart reviews. Overall, our estimates of *S. aureus*-SSTI hospitalization incidence was (~30%) higher than those of Klein, et al. [8], during the overlapping periods of the two studies. This difference may be due to the databases used (we used HCUP while Klein, et al., used the National Hospital Discharge Survey). In addition, we adopted a previous ICD-9 coding method [8] that included some diagnoses which are not universally classified as SSTIs, such as device infections and wound infections.

Another limitation is that the incidence of *S. aureus*-SSTI hospitalizations is only a fraction of all the burden of *S. aureus*-SSTIs in the population. For example, SSTIs are a major reason for emergency department (ED) visits [4,21]. Even though ED visits are associated with a lower cost than hospitalizations, they are high in volume, and may still account for a large national cost associated with *S. aureus*-SSTIs. Therefore, as suggested above, our methods could have led to an underestimate of total costs attributable to *S. aureus*-SSTIs. Finally, it should be noted that contemporary data from NIS are typically 3 years old making descriptions of current or past year trends impossible.

## Conclusions

In summary, between 2001 and 2009, the overall incidence of *S. aureus* hospitalizations increased by 57%, with the peak incidence in 2008. This increase was driven by *S. aureus*-SSTI hospitalizations, which rose by 105%. Children 0–17 years experienced the highest growth in *S. aureus*-SSTI hospitalizations (316%). However, the highest incidence of *S. aureus*-SSTI hospitalizations was observed in the population ≥ 75 years old across all the study years, and in 2009 the incidence in this age group was still about 7 times higher than in children. The national annual cost of *S. aureus*-SSTI hospitalizations increased by 26%. This increase could have been more dramatic if it were not for the counterbalancing shift in the demographics of *S. aureus*-SSTI hospitalizations to younger patients who have less expensive hospitalizations, and to the trend for shorter hospital stays for *S. aureus*-SSTIs. Efforts to prevent *S. aureus*-SSTIs and MRSA-associated SSTIs could have a meaningful impact on healthcare costs for this common infection.

## Competing interest

JS, HAM, AC, SB are employed by GlaxoSmithKline Group of Companies. As employees, JS, HAM and SB have restricted shares and AC has stock ownership in the GlaxoSmithKline Group of Companies. RM and PO are former employees of GlaxoSmithKline Group of Companies. LM has received consulting fees from GlaxoSmithKline, Durata Therapeutics and Pfizer.

## Authors’ contributions

Conception and design: JAS, RMM, AC, PO, HAM, SB, LGM; Acquisition and analysis of data: JAS, RMM; Interpretation of data: JAS, RMM, AC, PO, HAM, SB, LGM. Drafting of the manuscript: JAS, RMM, LGM; Critical revision of the manuscript for important intellectual content: JAS, RMM, AC, PO, HAM, SB, LGM. Statistical analysis: JAS, RMM; Obtaining funding: JAS; Administrative, technical, or material support: JAS; Supervision: JAS, RMM, LGM. All authors have given final approval of the version to be submitted.

## Pre-publication history

The pre-publication history for this paper can be accessed here:

http://www.biomedcentral.com/1471-2334/14/296/prepub

## References

[B1] Edelsberg J Taneja C Zervos M Haque N Moore C Reyes K Spalding J Jiang J Oster G Trends in US hospital admissions for skin and soft tissue infections Emerg Infect Dis 2009 14 1516 1518 10.3201/eid1509.081228 19788830 PMC2819854

[B2] Itani KM Merchant S Lin SJ Akhras K Alandete JC Hatoum HT Outcomes and management costs in patients hospitalized for skin and skin-structure infections Am J Infect Control 2011 14 42 49 10.1016/j.ajic.2010.03.018 20673598

[B3] Fridkin SK Hageman JC Morrison M Sanza LT Como-Sabetti K Jernigan JA Harriman K Harrison LH Lynfield R Farley MM Methicillin-resistant Staphylococcus aureus disease in three communities N Engl J Med 2005 14 1436 1444 10.1056/NEJMoa043252 15814879

[B4] Moran GJ Krishnadasan A Gorwitz RJ Fosheim GE McDougal LK Carey RB Talan DA Methicillin-resistant *S. aureus* infections among patients in the emergency department N Engl J Med 2006 14 666 674 10.1056/NEJMoa055356 16914702

[B5] Stevens DL Bisno AL Chambers HF Everett ED Dellinger P Goldstein EJ Gorbach SL Hirschmann JV Kaplan EL Montoya JG Wade JC Practice guidelines for the diagnosis and management of skin and soft-tissue infections Clin Infect Dis 2005 14 1373 1406 10.1086/497143 16231249

[B6] Naimi TS LeDell KH Boxrud DJ Groom AV Steward CD Johnson SK Besser JM O'Boyle C Danila RN Cheek JE Osterholm MT Moore KA Smith KE Comparison of community- and health care-associated methicillin-resistant Staphylococcus aureus infection JAMA 2003 14 2976 2984 10.1001/jama.290.22.2976 14665659

[B7] Hersh AL Chambers HF Maselli JH Gonzales R National trends in ambulatory visits and antibiotic prescribing for skin and soft-tissue infections Arch Intern Med 2008 14 1585 1591 10.1001/archinte.168.14.1585 18663172

[B8] Klein E Smith DL Laxminarayan R Hospitalizations and deaths caused by methicillin-resistant Staphylococcus aureus, United States, 1999–2005 Emerg Infect Dis 2007 14 1840 1846 10.3201/eid1312.070629 18258033 PMC2876761

[B9] Dryden M Complicated skin and soft tissue infections caused by methicillin-resistant Staphylococcus aureus: epidemiology, risk factors, and presentation Surg Infect (Larchmt ) 2008 14 Suppl 1 s3 10 18844473 10.1089/sur.2008.066.supp

[B10] Marton JP Jackel JL Carson RT Rothermel CD Friedman M Menzin J Costs of skin and skin structure infections due to Staphylococcus aureus: an analysis of managed-care claims Curr Med Res Opin 2008 14 2821 2828 10.1185/03007990802365169 18759996

[B11] Maragakis LL Perencevich EN Cosgrove SE Clinical and economic burden of antimicrobial resistance Expert Rev Anti Infect Ther 2008 14 751 763 10.1586/14787210.6.5.751 18847410

[B12] Menzin J Marton JP Meyers JL Carson RT Rothermel CD Friedman M Inpatient treatment patterns, outcomes, and costs of skin and skin structure infections because of Staphylococcus aureus Am J Infect Control 2010 14 44 49 10.1016/j.ajic.2009.04.287 19762120

[B13] Agency for Healthcare Research and Quality HCUP Nationwide Inpatient Sample (NIS). Healthcare Cost and Utilization Project (HCUP). 2001–2009 2012 http://www.hcup-us.ahrq.gov/nisoverview.jsp [serial online]

[B14] Certified Coding Specialist (CCS) preparation series Advance for health care professionals. Revisions address new ICD-9-CM codes, Part I 2008 http://health-information.advanceweb.com/Article/Revisions-Address-New-ICD-9-CM-Codes-Part-1.aspx [serial online]

[B15] United States Census Bureau National population estimates, 2001–2009 2012 http://www.census.gov/popest/data/historical/2000s/index.html

[B16] Agency for Healthcare Research and Quality HCUP Cost-to-Charge Ratio Files (CCR). Healthcare Cost and Utilization Project (HCUP). 2001–2009 2012 http://www.hcup-us.ahrq.gov/db/state/costtocharge.jsp [serial online]; Accessed September 12, 2012

[B17] Bureau of Labor Statistics website Consumer price index (CPI).CPI detailed report tables 2012 http://www.bls.gov/cpi/#tables

[B18] Mera RM Suaya JA Amrine-Madsen H Hogea CS Miller LA Lu EP Sahm DF O'Hara P Acosta CJ Increasing role of Staphylococcus aureus and community-acquired methicillin-resistant Staphylococcus aureus infections in the United States: a 10-year trend of replacement and expansion Microb Drug Resist 2011 14 321 328 10.1089/mdr.2010.0193 21417776

[B19] O’Hara FP Amrine-Madsen H Mera RM Brown ML Close NM Suaya JA Acosta CJ Molecular Characterization of Staphylococcus aureus in the United States 2004–2008 reveals the rapid expansion of USA300 among inpatients and outpatients Microb Drug Resist 2012 14 555 561 10.1089/mdr.2012.0056 22775581

[B20] Dantes R Mu Y Belflower R Aragon D Dumyati G Harrison LH Lessa FC Lynfield R Nadle J Petit S Ray SM Schaffner W Townes J Fridkin S National burden of invasive methicillin-resistant Staphylococcus aureus infections, United States, 2011 JAMA Intern Med 2013 14 1970 1978 24043270 10.1001/jamainternmed.2013.10423 PMC10887428

[B21] Pallin DJ Egan DJ Pelletier AJ Espinola JA Hooper DC Camargo CA Jr Increased US emergency department visits for skin and soft tissue infections, and changes in antibiotic choices, during the emergence of community-associated methicillin-resistant Staphylococcus aureus Ann Emerg Med 2008 14 291 298 10.1016/j.annemergmed.2007.12.004 18222564

